# Use of information entropy measures of sitting postural sway to quantify developmental delay in infants

**DOI:** 10.1186/1743-0003-6-34

**Published:** 2009-08-11

**Authors:** Joan E Deffeyes, Regina T Harbourne, Stacey L DeJong, Anastasia Kyvelidou, Wayne A Stuberg, Nicholas Stergiou

**Affiliations:** 1 Nebraska Biomechanics Core Facility, University of Nebraska at Omaha, Omaha, NE, 68182, USA; 2 Munroe-Meyer Institute, University of Nebraska Medical Center, Omaha, NE 68198, USA; 3 Department of Environmental, Agricultural and Occupational Health Sciences, College of Public Health, University of Nebraska Medical Center, Omaha, NE 68198, USA

## Abstract

**Background:**

By quantifying the information entropy of postural sway data, the complexity of the postural movement of different populations can be assessed, giving insight into pathologic motor control functioning.

**Methods:**

In this study, developmental delay of motor control function in infants was assessed by analysis of sitting postural sway data acquired from force plate center of pressure measurements. Two types of entropy measures were used: symbolic entropy, including a new asymmetric symbolic entropy measure, and approximate entropy, a more widely used entropy measure. For each method of analysis, parameters were adjusted to optimize the separation of the results from the infants with delayed development from infants with typical development.

**Results:**

The method that gave the widest separation between the populations was the asymmetric symbolic entropy method, which we developed by modification of the symbolic entropy algorithm. The approximate entropy algorithm also performed well, using parameters optimized for the infant sitting data. The infants with delayed development were found to have less complex patterns of postural sway in the medial-lateral direction, and were found to have different left-right symmetry in their postural sway, as compared to typically developing infants.

**Conclusion:**

The results of this study indicate that optimization of the entropy algorithm for infant sitting postural sway data can greatly improve the ability to separate the infants with developmental delay from typically developing infants.

## Background

Cerebral palsy, and other motor pathologies, give rise to altered patterns of movement. In order to quantify altered movement patterns in infants, postural sway during infant sitting can be analyzed for patterns using measures derived from information theory, such as approximate entropy and symbolic entropy. Measures such as these quantify patterns in time series data, making them potentially well suited for assessment of altered patterns of movement in a variety of movement pathologies, and may also provide insight into the nature of movement variability in human motor control pathologies [1-4].

Variability in control of human movement has historically been thought of in terms of error in a control system [5]. For example, if one is tossing darts, sometimes one might toss a bull's eye (meaning the dart goes in the very center of the circular pattern of the target), but the dart doesn't always go in the bull's eye because of variability in the motor control system. This leads some to the conclusion that a motor program was not executed correctly when the dart fails to go in the bull's eye, and from this perspective, variability is always an error in the motor control system. A more recent theory of motor control, based on dynamic systems theory, views the variability in motor control as part of the natural dynamics of the system [6]. Dynamic systems theory represents behaviors as being local minima on a potential surface, with the system proceeding towards a potential well like a marble rolling towards the bottom of a dish. Motor learning involves deepening the system's potential well associated with the behavior, and thus reducing variability. From this perspective, the potential well can never be infinitely deep, so there will always be some variability in the behavior. While a person tossing darts may wish for zero variability in their tosses, current theories on variability find that there are benefits to having some variability in movement. The theory of optimal movement variability focuses on the benefits of having a balance between rigid control and randomness in movement; i.e. complexity [7]. Having complexity in movement allows for exploration of new solutions to motor control in order to find optimal solutions. As stated by Hadders-Algra and colleagues, "Complexity points to the spatial variation of movements. It is brought about by the independent exploration of degrees of freedom in all body joints." [8,9]. Thus entropy, a measure of complexity from information theory, might be expected to differ in postural sway of infants with typical development, as compared to infants with motor development pathologies such as cerebral palsy.

The application of the concept of entropy to information theory has resulted in mathematical algorithms that are useful for describing randomness in experimental data from physiological systems. Information is a concept used in information theory, and is used in the sense that the string "ABABABABAB" has only a small amount of information in it (it is easy to guess what the next letter is – so the next letter adds no new information, hence a low information content) but "ABAABABABB" has more information (you could not determine for sure the next letter), even though both are strings of characters of the same length. Claude Shannon [10,11], developed the Shannon Entropy to describe the information content of a signal, with the idea that transmission of the signal for communication purposes needs to preserve the information content. If the goal of one's research is to characterize information in experimental physiologic time series, rather than in communication applications as Shannon did, there are some modifications that can be made to the algorithm. Perhaps the most widely used entropy measurement for experimental data from physiologic systems is the approximate entropy developed by Pincus [12]. The approximate entropy may serve as an indicator for the complexity of the underlying physiologic processes that give rise to the variability in the time series data [12]. In instances where pathology alters the complexity of the physiological process, the entropy value may serve as a means to identify the pathological state. For example, cardiac pathology may be identified by loss of complexity in heart rate data [13], concussions have been shown to cause loss of complexity in standing postural sway data [1], and knee ligament injury alters complexity in gait [14].

Other authors have developed different algorithms to assess entropy in experimental time series data [15-17], often with the goal of improving some aspect of the analysis. For example, one might desire to find a measure of randomness that does not depend on the length of the time series, i.e. the entropy should remain within a well defined range, regardless of the length of the time series. This would facilitate comparisons with data acquired in different laboratories, for example. Sample entropy has been used for this reason [15]. Both the approximate entropy and the sample entropy look at changes comparing patterns of length L with patterns of length L+1. Alternatively, the scaling of patterns at greatly different lengths, i.e. a pattern repeats but one repeat is longer or shorter than another, has been studied using multiscale entropy [16]. A data vector from time series data is a continuous subset of the list of numbers that comprise the time series data. Comparison of data vectors at different points along the time series is typically done by comparing the values, with similarity of the vectors being defined as one vector having values within a specified range of those in the comparison vector. However, comparison of the vectors can be performed using fuzzy logic, resulting in the fuzzy entropy [17], where the term "fuzzy" indicates that the similarity between the vectors is not a simple binary "yes" or "no", but rather the degree of similarity is calculated.

Different types of data may be best analyzed using different measures of complexity, and it is not clear *a priori *which type of analysis will be best for a particular type of data. For infant sitting postural sway data, approximate entropy has been used previously [18], but other methods have not been explored. For this work we have chosen to use the approximate entropy [12], the symbolic entropy [19], and asymmetric symbolic entropy, which is a modification of the symbolic entropy. While in our Methods section we provide more details on the algorithm, in short the symbolic entropy measures how much the infant's postural sway crosses certain locations on the force plate, called "threshold values". Typically only one threshold is used, the mean of the data. We modified the symbolic entropy algorithm to allow multiple threshold values to be used. These thresholds need not be symmetric – i.e. thresholds in one direction could be set differently from thresholds in the opposite direction in order to investigate asymmetry in the data. The use of two thresholds is motivated by the idea that the postural sway needs to be confined within the base of support to avoid a fall. Therefore control of posture near the center of the base of support might not be as critical as control of posture near the boundary. In order to investigate postural control near the boundaries of the base of support, two threshold values were used. Additionally, the use of different thresholds in the left and right directions allows the investigation of asymmetry of the postural sway, which can not be addressed with other measures of complexity.

Learning how to maintain upright sitting posture is an important motor developmental milestone. Infants use the upright sitting posture as a base from which to explore their immediate environment by reaching for nearby objects and to allow visual inspection of their immediate environment [20,21]. Additionally, sitting is important because it is one of first developmental milestones an infant achieves, and thus serves as an early indicator of the health of the motor control system [22]. The achievement of the sitting milestone is delayed in some pathologic populations, such as those with cerebral palsy. Identification of infants with delayed motor development at the youngest age possible is of interest because treatment early in life when neural plasticity is greatest may confer greater benefits. Some intervention methods for infants with cerebral palsy may prove better than others [23]. Quantifying the differences between various interventions using sitting postural sway will assist researchers evaluating the various interventions. Specifically, cerebral palsy is a multifaceted pathology, and there is great variability in the pathology among the affected population [24]. Thus what works best for one infant may not be optimal for another infant. Early evaluation of the effectiveness of one intervention may allow early change of treatment, while neural plasticity is still greatest. For example, if an infant is found to not be responding to a particular intervention, an alternative could be implemented as soon as the first intervention can be determined to not be optimal. Thus, use of sitting postural sway as an early window into the developing motor control system could have potential clinical benefits.

While being able to extract information about the infant's motor control capabilities from sitting postural sway data could be beneficial, the best analytical method to do so has not yet been identified. Linear measures, such as standard deviation or range of sway, may be used to describe how much movement there is in the postural sway. However, the complexity of the movements that an infant makes may be a better predictor of pathology that simply how much movement [9]. The entropy measures discussed above are promising because they have been developed to assess the complexity of a time series, rather than just assessing the amount of movement. We anticipate that the complexity of the postural sway will give insight into the motor control pathology in cerebral palsy, as it has in other motor control studies, including concussion [1], grip force in Parkinson's disease [2], stereotypical rocking in severe retardation [3], and loss of visual/cutaneous feedback [4]. However, the best algorithm to use for infant sitting needs to be determined. The reason for comparing different parameter values is to understand the impact of parameter choice on the outcome of the analysis, as different researchers will use different parameters in their analysis. But more importantly, in order for a measure to be clinically useful, it needs to maximize the ability to classify individuals correctly into one population or the other. The approach used here was to examine t-scores, the statistic used in the independent t-test to compare two populations, with the goal of maximizing the ability of the algorithm to separate the two populations.

Therefore, the goal of this investigation was to determine the utility of several different entropy algorithms in differentiating between sitting posture data of infants who have typical motor skills from sitting posture data of infants who have delayed development of motor skills. We hypothesized that infants with developmental delay will have altered complexity of postural control, because optimal variability theory suggests that pathology can be associated with either higher or lower complexity of movement [7]. Further, we hypothesized that asymmetric measures of postural control will vary in the infants with developmental delay as compared to typically developing infants in the anterior-posterior direction (forward-backwards direction), since falling forward results in a soft landing on the legs, but falling backwards needs to be more carefully controlled.

## Methods

### Subjects

Infants were recruited into the study when they were just developing the ability to sit upright, and all infants participated for several months. However, the data used for this analysis is only from the last session for each infant, so it represents the most mature sitting behavior that was collected for each infant. Recruitment was done through newsletters, flyers, and pediatric physical therapists employed at the University. Twenty-two developmentally delayed infants, age 11.97 months to 27.8 months (mean = 17.70, std = 3.93); and nineteen typically developing infants, age 7.03 to 9.8 months (mean = 8.13, std = 0.71) participated in the study. Infants in the developmentally delayed group were diagnosed with cerebral palsy, or else were developmentally delayed and at risk for cerebral palsy. At risk infants met one or more of the following conditions: premature delivery, brain bleeding (of any level of severity), diagnosis of periventricular leukomalacia, or significantly delayed gross motor development as measured on standardized testing. Because a definitive diagnosis of cerebral palsy could not been made by our collaborating physicians, we refer to these infants as developmentally delayed, and all scored below 1.5 standard deviations below the mean for their corrected age on the Peabody Gross Motor Scale [25]. Exclusion criteria included having an untreated, diagnosed visual impairment, a diagnosed hip dislocation or subluxation greater than 50%, or an age outside the range 5 months to 24 months at the start of the study, which was 4 months prior to the data collection session used for this analysis. Typically developing infants were screened for normal development by a physical therapist prior to admission into the study, being excluded if they failed to score above 0.5 standard deviations below the mean on the Peabody Gross Motor Scale, had a diagnosed visual impairment, had a diagnosed musculoskeletal problem, or were older than five months at the start of the study. A consent form was signed by a parent of all infant participants, and all procedures were approved by the University of Nebraska Medical Center Institutional Review Board.

### Data collection

**Figure 1:**
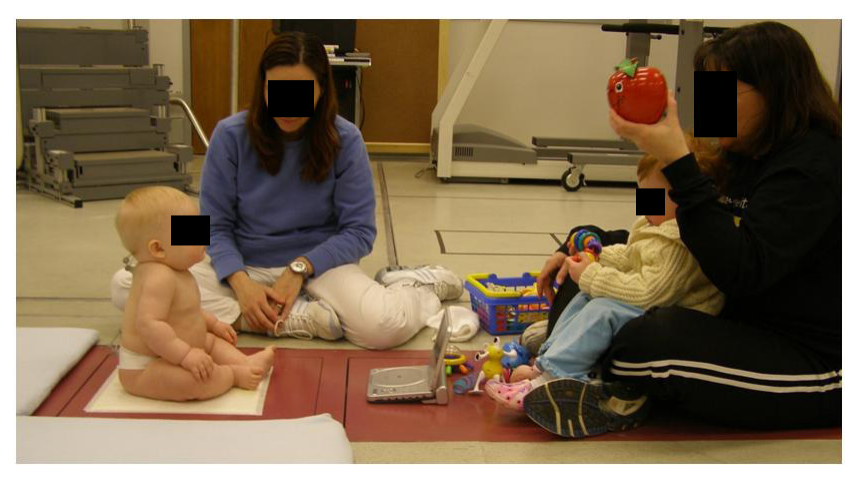
**Infant sits on force platform for data collection, with researcher and parent near by**.

For data acquisition, infants sat on an AMTI force plate (Watertown, MA), interfaced to a computer system running Vicon data acquisition software (Lake Forest, CA). Center of Pressure (COP) data were acquired through the Vicon software at 240 Hz, in order to be above a factor of ten higher than the highest frequency that contained relevant signal as established via spectral analysis from pilot work. Segments of usable (described below) data were analyzed using custom MatLab software (MathWorks, Nantick, MA). No filtering was performed in order to not alter the entropy results [26]. Trunk and pelvis markers were also placed on the infant, but the marker data was not analyzed for this study. An assistant sat to the left side of the infant during data acquisition, and a parent or relative (typically the mother) sat in front of the infant, for comfort and support, as well as to keep the infant's attention focused on toys held in front of the infant (Fig. 1).

Trials were recorded including force plate data and video data from the back and side views. Afterwards segments were selected by viewing the corresponding video. Segments of data with 2000 time steps (8.3 seconds at 240 Hz) were selected from these trials by examination of the video. The COP data allows medial-lateral (side-to-side) and anterior-posterior (front to back) to be analyzed separately. Acceptable segments were required to have no crying or long vocalization, no extraneous items (e.g. toys) on the force platform, neither the assistant nor the mother were touching the infant, the infant was not engaged in rhythmic behavior (e.g. flapping arms), and the infant had to be sitting and could not be in the process of falling.

### Data analysis

#### Symbolic entropy

Calculation of symbolic entropy was performed on postural sway data in both the medial-lateral movement, and in the anterior-posterior movement, using the methodology presented by Aziz and Arif [19]. It is a four step process:

1. Convert the time series into a binary symbol series based on a threshold value. Time series data points below the threshold are replaced by 0, those above the threshold value are replaced by 1.



**Figure 2:**
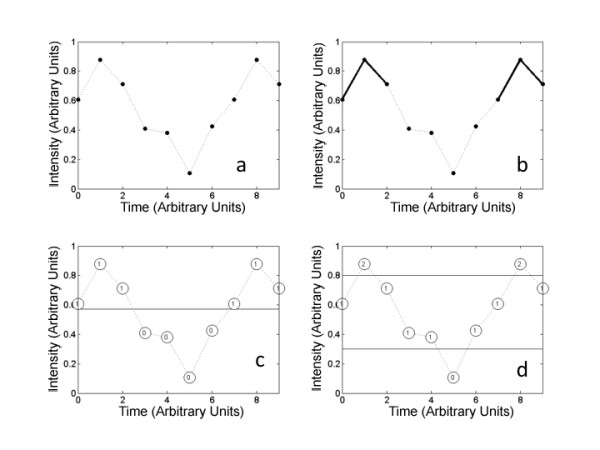
**Entropy calculations**. Entropy calculations: A. time series data. B. Approximate entropy counts similar vectors; here two similar vectors are shown in bold. C. Symbolic entropy with one threshold creates a time series based on whether a point is above or below the mean. Note that the value changes as the time series crosses the threshold. D. Two thresholds allow sensitivity to movement that is not close to the center, and thus closer to the presumed edge of the base of support.



With a threshold of 0.5718 (mean of the data) is converted to the following symbol series:



2. Words are formed from the symbols, each with a word length L. For our example, using a word length of three:



that is then represented as a word series (Fig 2c):

3. The word series can be transformed by conversion of the binary into decimal: (000 = 0, 001 = 1, 010 = 2, 011 = 3, 100 = 4, 101 = 5, 110 = 6, 111 = 7) into a word symbol series:



4. Shannon's entropy can be calculated from this word symbol series, and then corrected and normalized as described by Aziz and Arif [19]. However, it is this process of conversion to a symbolic time series that is critical in finding relevant patterns in the time series.

The threshold value is a key aspect of the process, as points in the time series are either above or below the threshold value. Selection of too low of a threshold produces more ones than zeros, with a correspondingly high number of words with mostly ones. Conversely selecting too high of a threshold value results in more zeros in the symbol series, with a correspondingly high number of words with mostly zeros. If the symbol series is mostly ones (or mostly zeros) then the corresponding entropy will be low, and the complexity of the time series will not be appropriately captured in the result. Thus selection of a threshold value must be done carefully. One method is to select the mean value for the time series, thereby ensuring that half of the symbols will be zeros and half will be ones, as was done by Aziz and Arif [19]. As an example, consider the analysis with a word length of three. The words that are encoded with this approach will have a value 0 (000) if the infant stays on the low side of the mean for the time interval that corresponds to that word; or a value of 7 (111) if the infant stays on the high side of the mean for the time interval that corresponds to that word. The only way the word will have a value of other than 0 or 7 will be if the infant moves past the average value during the time interval that corresponds to that particular word. The entropy value calculated with this approach will then be a reflection of the movement back and forth past this mean value. The important question is whether this reflects a clinically meaningful measure or not.

Control of the system near the average value may not be the most sensitive measure of physiologic function of the postural control system. It may be that control towards the extreme values of postural sway, where there is a greater likelihood of falling over, would be more diagnostic of pathology in neuromuscular control. With just a single threshold value in the symbolic entropy, this can not really be explored fully. Thus a second method of calculating the symbolic entropy was devised with two threshold values. Choosing values of 0.3 and 0.8 for the threshold values, the time series



is converted to the symbol series (Fig. 2d):



where 0 indicates a data point below the lower threshold, 2 indicates a data point above the upper threshold, and 1 indicates a data point in between the thresholds. Again, using a word length of three for this example, the following words are obtained:



with a word length of three and three symbols possible, there are 3^3 = 27 possible words, coded from 0 to 26 as follows:



So that the word series formed is:



As with the single threshold symbolic entropy, Shannon's entropy is calculated from the word series, and then the normalized corrected Shannon's entropy is calculated.

The thresholds in all cases were based on the mean value of each time series, and new threshold values were calculated for each time series. In some cases of multiple thresholds, the thresholds were determined from the standard deviation of the time series. The strategy in these calculations is to examine a movement at each time step as it relates to the overall movement in that time series. In other cases, the thresholds were set as a certain number of millimeters above or below the mean. The strategy in these calculations is to examine at the actual distance moved in millimeters at each time step. In most cases the thresholds were set symmetrically, with the same distance above and below the mean being used. However, a few non-symmetric thresholds were also investigated. For example, 0 might be assigned to data points below minus three standard deviations, 1 assigned to data points between minus three standard deviations and plus one standard deviations, and 2 assigned to all data points above one standard deviation. In this example, excursions have to be three standard deviations away from the mean in the left direction, but only one standard deviation in right direction, to trigger the assignment of a different symbol. Once the symbols have been assigned, the Shannon entropy is calculated, and then normalized, as was done for the symbolic entropy, using the method of Aziz and Arif [19]. The entire procedure is performed twice, once for data from the anterior-posterior direction, and once for the data from the medial-lateral direction.

#### Approximate entropy

The approximate entropy (ApEn) was calculated using MatLab code developed by Kaplan and Staffin [27], implementing the methodology of Pincus [12]. Approximate entropy is a measure of how disorderly a time series is [12] and can be used to assess disorderliness in movement when applied to COP time series data. The general strategy in the calculation of approximate entropy is to examine all the points in the data set for short pattern repeats (Fig. 2a). The length of the repeat pattern is defined by a parameter m. This is done by using a vector of length m starting at point p_i_, and then counting how many other vectors at other points p_j _(j ≠ i) in the time series have a similar pattern, repeating the procedure for all vectors of length m in the time series, and summing the logarithm of the results. The r parameter defines how similar a second vector has to be in order to be counted. Another parameter, lag, indicates how many time steps there are between points in one of the length m vectors. For example, if lag = 1, then adjacent points are used. To calculate approximate entropy, the log of this similarity count is normalized by the number of points in the time series. Thus three parameters are used in this algorithm, m, r, and lag. Typical values for biomechanics data analysis are lag = 1, r = 0.2 to 0.25 times the standard deviation of the time series, and m = 2 [2,28,29].

### Statistical analysis

One goal of the statistical analysis was to find the best entropy measure to separate the two populations, since the entropy measure identified in this manner would presumably have the best chance of having clinically useful sensitivity to changes in postural control with physical therapy interventions, a long range goal of this research. In order to assess the effectiveness in separating the two populations (delayed versus typical development), we used the t-score, which is a measure of the separation between the two populations relative to the variances of the populations. The t-scores, also called t-statistics or t-values that are commonly used in independent t-tests [30], were calculated by dividing the difference in means between the two populations (mean of delayed development minus mean of typically developing) by the root mean square of the standard deviations, for each set of parameters used for each type of entropy, for COP data from both anterior-posterior and medial-lateral directions. A negative sign on the t-score indicates that the mean of the data from the typically developing is larger than the mean of the data from delayed development. The t-score indicates how much the two populations overlap for the given measure, with larger magnitude indicating less overlap.

The analysis includes multiple comparisons, but they are not all independent. In other words, the entropy calculated with one set of parameters is correlated with the entropy calculated with a slightly different set of parameters, and values of t scores in the tables 1, 2, 3 and 4 are similar to values nearby. We have 2 types of entropy (approximate entropy and symbolic entropy) and 3 parameters for each (approximate entropy has m, r, and lag; symbolic entropy has number of threshold values, position of threshold, and symmetry of thresholds). Thus, there are 2 times 3 equal with 6 parameters that we have adjusted independently. This number times 2 (for postural sway in the two directions: the anterior-posterior and medial-lateral) gives a total of 12. The Bonferroni correction requires the p-value to be adjusted for the number of independent comparisons. Thus, we set the *p*_*crit *_= .05/12 = .00417, corresponding to a t-score of magnitude 3.04 for a t-tailed test with 39 degrees of freedom (dof = n_1 _+ n_2 _– 2; where n_1 _and n_2 _are the number of subjects sampled from the two populations).

**Table 1:** Symbolic entropy t-scores for comparison of medial-lateral postural sway

	Word length used in symbolic entropy calculation									
One threshold	1	2	3	4	5	6	7	8	9	10
	
M	-0.93	-0.68	0.77	-1.85	-1.44	-1.40	-1.13	-1.05	-1.12	-1.18
										
Two thresholds										
m - .01 std, m + .01 std	-1.20	-1.61	-1.62	-1.47	-1.39	-1.25	-1.24	-1.22	-1.31	-1.33
m - .1 std, m + .1 std	-1.26	-0.32	-0.41	-0.71	-0.72	-0.88	-1.07	-1.23	-1.30	-1.31
m - .2std, m + .2std	-0.48	-0.86	-0.67	-1.19	-1.35	-1.53	-1.46	-1.42	-1.32	-1.21
m - .5 std, m + .5 std	0.37	-1.23	-1.15	-0.51	-0.61	-0.77	-0.84	-1.03	-1.13	-1.21
m - 1 std, m + 1 std	0.44	0.29	-0.53	-1.70	-1.98	-2.10	-1.86	-1.64	-1.38	-1.22
m - 2 std, m + 2 std	-0.61	-1.07	-1.15	-0.71	-0.49	-0.43	-0.39	-0.36	-0.33	-0.31
m - 2.5 std, m + 2.5 std	-1.13	-1.04	-1.20	-1.13	-0.93	-0.82	-0.77	-0.76	-0.75	-0.77
m - 2.8 std, m + 2.8 std	-0.98	-1.30	-1.52	-1.70	-1.95	-1.99	-2.01	-2.02	-2.00	-1.97
m - 2.9 std, m + 2.9 std	-0.97	-1.38	-1.66	-1.74	-1.81	-1.82	-1.84	-1.92	-2.00	-2.05
m - 3 std, m + 3 std	-2.68	-2.76	-2.57	-2.36	-2.52	-2.59	-2.64	-2.68	-2.71	-2.79
m - 3.1 std, m + 3.1 std	-2.31	-2.67	-2.85	-2.85	-2.73	-2.62	-2.55	-2.56	-2.59	-2.62
m - 3.2 std, m + 3.2 std	-1.56	-1.92	-2.16	-2.24	-2.30	-2.32	-2.31	-2.31	-2.34	-2.35
m - 3.5 std, m + 3.5 std	-2.10	-2.24	-2.25	-2.24	-2.25	-2.25	-2.25	-2.26	-2.27	-2.29
m - 1 mm, m + 1 mm	-0.34	-1.79	-1.85	-1.69	-1.11	-1.08	-1.18	-1.34	-1.41	-1.45
m - 10 mm, m + 10 mm	-0.30	-0.49	-0.25	-0.17	-0.30	-0.46	-0.57	-0.64	-0.67	-0.67
m - 15 mm, m + 15 mm	0.61	0.59	0.42	0.19	0.06	-0.05	-0.04	-0.03	0.00	0.04
m - 20 mm, m + 20 mm	0.64	0.65	0.58	0.59	0.60	0.57	0.54	0.54	0.55	0.55
m - 25 mm, m + 25 mm	-0.39	-0.53	-0.39	-0.38	-0.30	-0.26	-0.27	-0.28	-0.29	-0.32
m - 22 mm, m+ 22 mm	-0.40	-0.53	-0.52	-0.54	-0.51	-0.45	-0.47	-0.47	-0.47	-0.50
m - 30 mm, m + 30 mm	-0.07	-0.14	0.14	0.43	0.48	0.50	0.51	0.50	0.48	0.46
m - 35 mm, m + 35 mm	0.30	0.46	0.65	0.77	0.82	0.84	0.85	0.85	0.84	0.83
m - 40 mm, m + 40 mm	0.22	0.45	0.65	0.77	0.82	0.82	0.82	0.81	0.80	0.79
m - 2 std, m + 3 std (A)	-1.30	-1.40	-1.20	-0.86	-0.73	-0.63	-0.60	-0.62	-0.65	-0.68
m - 1std, m + 3 std (A)	-1.39	-1.54	-1.45	-1.04	-1.07	-1.06	-0.95	-0.81	-0.67	-0.63
m - 3 std, m + 2 std (A)	-1.86	-2.19	-2.28	-1.85	-1.57	-1.46	-1.34	-1.22	-1.13	-1.08
m - 3 std, m + 1 std (A)	-2.52	-2.64	-2.61	-3.33*	-3.42*	-3.48*	-3.05*	-2.68	-2.28	-1.99
										
Three thresholds										
m - .01 std, m, m + .01 std	-1.16	-1.77	-2.23	-2.76	-2.25	-1.20	-0.72	-1.05	-1.14	-1.85
m - .1 std, m, m + .1 std	-1.49	0.91	-1.11	-1.16	-2.50	-2.15	-1.47	-2.08	-2.77	-1.60
m - .2std, m, m + .2std	-2.67	-1.38	-1.43	-0.54	0.57	0.64	-0.58	-0.58	-0.19	0.43
m - .5 std, m, m + .5 std	-0.27	0.19	0.15	-1.13	-1.33	-1.51	-1.91	-2.51	-1.69	-0.70
m - 1 std, m, m + 1 std	-0.18	-0.31	-0.60	-1.30	-0.68	-0.93	-0.63	-1.11	-2.70	-2.17
m - 2 std, m, m + 2 std	-2.89	-2.58	-2.35	-2.66	**-3.07**	-2.29	-1.57	-0.61	-0.37	0.10
m - 2.5 std, m, m + 2.5 std	-2.24	-1.45	-0.95	-1.41	-1.24	-1.40	-0.99	-1.33	-2.59	-2.21
m - 2.8 std, m, m + 2.8 std	-1.32	-1.05	-0.92	-1.16	-1.71	-1.46	-1.64	-1.57	-1.71	-1.53
m - 2.9 std, m, m + 2.9 std	-1.62	-1.44	-1.54	-1.54	-1.62	-1.51	-1.53	-2.37	-1.37	-1.04
m - 3 std, m, m + 3 std	-1.25	-0.96	-1.04	-1.50	-1.16	-1.67	-2.09	**-3.06**	-1.90	-1.42
m - 3.1 std, m, m + 3.1 std	-1.32	-0.94	-1.21	-1.24	-1.09	-1.01	-1.04	-1.15	-1.08	-1.22
m - 3.2 std, m, m + 3.2 std	-1.02	-1.26	-1.55	-2.10	-1.52	-1.46	-1.07	-1.46	-1.41	-1.12
m - 3.5 std, m, m + 3.5 std	-2.04	-1.74	-1.68	-1.63	-1.15	-0.69	-1.28	-1.34	-1.05	-0.89
m - 1 mm, m, m + 1 mm	0.80	0.88	1.68	1.73	1.15	0.67	0.96	0.37	0.24	-0.13
m - 10 mm, m, m + 10 mm	-1.24	-2.08	-2.20	-1.86	0.91	-0.22	0.24	0.92	1.43	1.48
m - 15 mm, m, m + 15 mm	0.41	0.57	1.52	-0.09	-0.21	-1.07	-0.55	-0.54	-0.92	-2.06
m - 20 mm, m, m + 20 mm	0.49	1.46	1.76	1.45	1.28	0.41	1.21	0.90	0.95	0.96
m - 25 mm, m, m + 25 mm	1.80	0.55	1.04	1.76	0.70	0.80	0.85	1.24	0.55	0.82
m - 22 mm, m, m+ 22 mm	-0.03	-1.25	-0.57	-0.45	-0.76	-1.78	-1.50	-1.21	1.63	0.61
m - 30 mm, m, m + 30 mm	1.26	0.59	1.09	0.97	1.00	1.11	0.83	-0.41	-0.27	-1.44
m - 35 mm, m, m + 35 mm	0.06	0.48	1.04	1.73	1.04	0.52	0.62	1.14	1.02	0.55
m - 40 mm, m, m + 40 mm	-0.23	-0.20	-0.21	-0.12	0.81	0.17	0.75	0.68	0.14	0.64
m - 2 std, m, m + 3 std (A)	1.26	0.80	0.62	1.15	1.04	0.79	1.00	0.92	0.90	1.01
m - 1std, m, m + 3 std (A)	0.85	0.37	0.75	0.61	0.26	0.84	1.30	1.84	1.33	0.91
m - 3 std, m, m + 2 std (A)	-0.37	-0.12	0.65	0.65	0.61	0.64	0.64	0.66	0.92	0.42
m - 3 std, m, m + 1 std (A)	1.08	0.94	1.09	1.02	0.99	1.06	1.45	0.02	-0.25	0.13

t-scores for comparison of medial-lateral postural sway of infants with typical development and with delayed development, based on symbolic entropy calculated with various thresholds and word lengths, -3.48 is the largest magnitude t-score.

Note: t-scores with magnitude equal or larger than 3.04 are indicated with * and are in bold. The "m" indicates mean value for the time series, "std" indicates the standard deviation for the time series, and "mm" indicates millimetres of movement in the COP. (A) indicates asymmetric thresholds were used

**Table 2:** Symbolic entropy t-scores for comparison of anterior-posterior postural sway

	Word length used in symbolic entropy calculation									
One threshold	1	2	3	4	5	6	7	8	9	10
	
M	0.67	1.63	1.48	0.83	0.68	0.52	0.69	0.80	0.91	0.95
										
Two thresholds										
m - 1 std, m + 1 std	0.34	1.01	1.27	0.86	0.25	-0.13	-0.20	-0.25	-0.25	-0.23
m - .5 std, m + .5 std	-0.25	1.17	0.51	0.11	-0.21	-0.42	-0.13	0.15	0.31	0.40
m - .2std, m + .2std	1.53	1.24	1.12	1.01	1.20	1.14	1.04	0.98	0.97	1.02
m - .1 std, m + .1 std	1.67	0.52	0.80	0.86	1.08	1.31	1.62	1.79	1.87	1.92
m - .01 std, m + .01 std	1.32	0.41	0.75	0.53	0.72	0.85	0.96	1.12	1.25	1.32
m - 2 std, m + 2 std	0.94	1.24	1.54	1.36	0.99	0.47	0.27	0.17	0.11	0.07
m - 2.5 std, m + 2.5 std	0.38	0.80	1.17	1.51	1.52	1.39	1.35	1.32	1.37	1.43
m - 3 std, m + 3 std	0.21	0.54	0.93	1.16	1.16	1.13	1.09	1.07	1.05	1.01
m - 3.5 std, m + 3.5 std	-0.16	-0.07	0.01	0.12	0.20	0.26	0.31	0.29	0.29	0.30
m - 2.8 std, m + 2.8 std	0.98	0.89	0.90	0.94	1.04	1.08	1.08	1.09	1.11	1.16
m - 3.2 std, m + 3.2 std	0.25	0.36	0.55	0.69	0.77	0.76	0.77	0.78	0.78	0.77
m - 3.1 std, m + 3.1 std	0.02	0.21	0.60	0.84	0.81	0.78	0.76	0.70	0.69	0.67
m - 2.9 std, m + 2.9 std	0.22	0.38	0.65	0.86	1.01	1.03	1.03	1.01	0.98	0.97
m - 1 mm, m + 1 mm	1.63	1.29	1.15	1.40	1.32	1.21	1.06	0.98	1.01	1.09
m - 10 mm, m + 10 mm	-0.60	-0.28	-0.47	-0.56	-0.58	-0.67	-0.70	-0.73	-0.73	-0.74
m - 15 mm, m + 15 mm	-0.74	-0.36	-0.19	-0.20	-0.45	-0.74	-0.87	-0.97	-1.08	-1.18
m - 20 mm, m + 20 mm	-1.33	-1.19	-1.27	-1.39	-1.48	-1.57	-1.66	-1.69	-1.66	-1.66
m - 25 mm, m + 25 mm	-0.94	-0.74	-0.89	-0.89	-0.94	-0.99	-1.02	-1.01	-1.03	-1.03
m - 22 mm, m+ 22 mm	-0.81	-0.69	-0.68	-0.69	-0.77	-0.80	-0.85	-0.91	-0.95	-0.98
m - 30 mm, m + 30 mm	-1.40	-1.12	-1.14	-1.20	-1.22	-1.25	-1.27	-1.30	-1.30	-1.31
m - 35 mm, m + 35 mm	-2.03	-2.08	-2.11	-2.13	-2.13	-2.13	-2.12	-2.12	-2.12	-2.12
m - 40 mm, m + 40 mm	-2.13	-2.15	-2.13	-2.11	-2.09	-2.07	-2.07	-2.06	-2.06	-2.05
m - 2 std, m + 3 std	0.93	1.29	1.58	1.53	1.16	0.79	0.65	0.62	0.61	0.58
m - 1std, m + 3 std	-0.02	-0.07	0.25	0.51	0.31	-0.06	-0.11	-0.18	-0.26	-0.37
m - 3 std, m + 2 std	0.16	0.40	0.73	0.80	0.82	0.56	0.36	0.20	0.05	-0.02
m - 3 std, m + 1 std	0.54	1.08	1.50	1.53	1.04	0.66	0.42	0.29	0.27	0.34
										
Three thresholds										
m - 1 std, m, m + 1 std	-0.95	-1.58	-2.34	-0.94	-0.59	-0.50	0.51	0.60	-0.59	-0.60
m - .5 std, m, m + .5 std	-0.53	-0.98	-1.46	-0.68	-1.09	-1.04	-2.76	-2.25	-1.29	-1.93
m - .2std, m, m + .2std	0.43	-1.09	-1.40	-1.70	-2.21	-2.88	-1.63	-0.99	-0.37	-0.96
m - .1 std, m, m + .1 std	-1.18	-0.23	0.45	0.61	-0.33	-0.45	0.22	0.74	0.74	-0.65
m - .01 std, m, m + .01 std	-1.01	-1.40	-2.76	-2.81	-2.02	-2.73	-3.27*	-2.12	-1.38	-0.36
m - 2 std, m, m + 2 std	-1.61	-1.67	-0.78	-0.40	-0.40	-1.65	-1.12	-1.83	-2.06	-3.29*
m - 2.5 std, m, m + 2.5 std	-2.28	-2.37	-2.66	-2.15	-1.70	-1.13	-0.90	-0.43	-1.64	-1.70
m - 3 std, m, m + 3 std	-0.99	-1.49	-1.31	-1.13	-0.94	-1.22	-2.02	-1.68	-1.89	-1.82
m - 3.5 std, m, m + 3.5 std	-0.95	-1.18	-1.05	-1.41	-1.78	-2.46	-1.68	-1.47	-1.02	-1.50
m - 2.8 std, m, m + 2.8 std	-1.69	-2.01	-1.22	-0.79	-1.16	-1.20	-1.01	-0.89	-0.93	-1.09
m - 3.2 std, m, m + 3.2 std	-0.97	-1.17	-1.64	-1.43	-1.57	-1.54	-1.65	-1.51	-1.61	-2.30
m - 3.1 std, m, m + 3.1 std	-1.49	-1.89	-1.44	-1.45	-1.11	-1.42	-1.43	-1.18	-1.03	-1.20
m - 2.9 std, m, m + 2.9 std	-1.26	-1.29	-1.16	-1.10	-1.12	-1.21	-1.15	-1.23	-1.47	-1.76
m - 1 mm, m, m + 1 mm	1.03	0.30	0.06	0.24	1.73	-0.38	-0.53	-1.19	-0.75	-0.61
m - 10 mm, m, m + 10 mm	1.20	0.98	0.30	1.56	1.39	0.98	0.74	0.15	1.08	0.57
m - 15 mm, m, m + 15 mm	-2.07	-1.73	1.43	0.14	0.59	1.34	1.21	1.20	1.02	0.92
m - 20 mm, m, m + 20 mm	0.87	-0.23	0.01	-1.07	-0.58	-0.42	-0.75	-2.00	-1.75	-1.61
m - 25 mm, m, m + 25 mm	1.49	1.60	1.41	0.49	1.15	0.97	1.11	1.10	0.88	-0.38
m - 22 mm, m, m+ 22 mm	1.06	1.53	0.30	0.58	0.89	1.51	0.88	0.67	1.09	1.44
m - 30 mm, m, m + 30 mm	-0.45	-0.46	-0.50	-0.66	-0.62	-0.40	1.19	0.40	1.04	1.03
m - 35 mm, m, m + 35 mm	0.93	0.79	0.82	0.95	1.05	-0.52	-0.62	-1.55	-0.29	-0.33
m - 40 mm, m, m + 40 mm	1.18	1.80	1.14	0.69	0.59	1.14	1.03	0.70	0.97	0.86
m - 2 std, m, m + 3 std (A)	1.30	-0.31	-0.52	-0.59	0.43	0.42	0.41	0.45	0.45	0.47
m - 1std, m, m + 3 std (A)	0.69	1.22	1.08	0.90	1.07	1.00	0.98	1.06	1.39	-0.12
m - 3 std, m, m + 2 std (A)	0.79	0.70	0.32	0.86	1.37	1.82	1.37	0.95	0.71	1.24
m - 3 std, m, m + 1 std (A)	0.74	0.74	0.69	0.72	0.72	0.73	0.93	0.43	0.81	0.77

t-scores for comparison of anterior-posterior postural sway of infants with typical development and with delayed development, based on symbolic entropy calculated with various thresholds and word lengths, -3.29 is the largest magnitude t-score.

Note: t-scores with magnitude equal or larger than 3.04 are indicated with * and are in bold. The "m" indicates mean value for the time series, "std" indicates the standard deviation for the time series, and "mm" indicates millimetres of movement in the COP. **(A) indicates asymmetric thresholds were used**

**Table 3:** Approximate entropy t-scores for comparison of medial-lateral postural sway

r value used in ApEn calculation												
m	lag	0.05*std	0.1*std	0.2*std	0.4*std	0.8*std	1.5*std	2.5*std	3*std	3.5*std	4*std	5*std
		
2	1	-0.94	-0.55	-0.46	-0.47	-0.56	-0.67	-0.20	-0.26	-1.14	-2.12	-0.76
4	1	0.58	-1.08	-1.22	-1.20	-1.37	-1.67	-1.62	-1.40	-2.26	-3.17*	-2.04
8	1	1.05	-0.14	-0.63	-1.69	-1.92	-2.40	-2.52	-2.54	-2.88	-3.27*	-2.69
2	4	-1.26	-1.41	-1.94	-2.46	-2.72	-2.68	**-3.09**	-3.32*	-3.27*	-3.17*	-2.04
4	4	1.23	-0.17	-1.55	-2.41	-2.84	-2.81	**-3.07**	-3.24*	-3.20*	-3.10*	-1.67
8	4	1.34	0.33	0.16	-2.39	-2.64	-2.64	-2.49	-2.93	-3.16*	-3.13*	-1.32
2	8	-1.32	-1.50	-2.18	-2.72	-2.82	-2.71	-3.02	-3.16*	**-3.08**	-2.90	-1.54
4	8	1.64	0.46	-1.51	-2.68	-2.60	-2.47	-2.45	-2.86	-3.03	-2.91	-1.15
8	8	1.35	0.50	1.29	-1.96	-2.91	-2.06	-1.96	-2.20	-2.49	-2.83	-1.73

t-scores for comparison of medial-lateral postural sway of infants with typical development and with delayed development, based on approximate entropy calculated with various lag and r values, -3.32 is the largest magnitude t-score.

Note: t-scores with magnitude equal or larger than 3.04 are indicated with * and are in bold.

**Table 4:** Approximate entropy t-scores for comparison of anterior-posterior postural sway

r value used in ApEn calculation												
m	lag	0.05*std	0.1*std	0.2*std	0.4*std	0.8*std	1.5*std	2.5*std	3*std	3.5*std	4*std	5*std
		
2	1	0.83	0.82	0.84	0.99	0.99	1.03	0.92	1.46	1.14	0.54	0.69
4	1	0.50	0.17	0.25	0.60	0.61	0.73	0.36	0.87	0.59	0.28	0.12
8	1	-1.04	0.68	0.41	0.28	0.24	0.40	-0.19	0.53	0.30	0.22	0.17
2	4	0.61	0.60	0.46	0.16	0.02	0.40	-0.30	0.41	0.23	0.24	0.04
4	4	1.15	1.05	0.84	0.48	0.17	0.17	-0.38	0.39	0.24	0.31	0.20
8	4	-0.80	0.55	1.03	1.01	0.39	0.49	-0.48	0.44	0.12	0.36	0.46
2	8	1.27	1.01	0.90	0.36	0.10	0.21	-0.33	0.39	0.25	0.35	0.17
4	8	0.15	1.26	1.09	0.90	0.36	0.54	-0.42	0.32	0.18	0.42	0.39
8	8	-1.04	-0.49	0.90	1.47	0.85	0.34	-0.05	0.21	0.20	0.34	0.43

t-scores for comparison of anterior-posterior postural sway of infants with typical development and with delayed development, based on approximate entropy calculated with various m, lag and r values, are all lower than 3.04.

Note: No t-scores with magnitude equal or larger than 3.04 are in this table.

## Results

The t-score results (Table 1) indicated that the symbolic entropy does find significant differences between the medial-lateral postural sway of typically developing infants compared to infants with delayed development. The t-score results in the anterior-posterior direction were less able to detect separation between the two populations (Table 2). The largest t-scores are for two threshold analysis with non-symmetric thresholds, as presented in last row of two-threshold analyses in Table 1. The larger magnitude t-scores (Table 1) are connected with two threshold values being assigned relatively far away from the mean, with the thresholds assigned on the order of three standard deviations above and below the mean value of the COP. This is consistent with the notion that control near the extreme positions (i.e. far to the right or far to the left) is important, since poor control near the extreme values of the COP may result in a fall. The best threshold of those tested was the mean-3 std, mean+1 std. This means that excursions farther away from the mean to the left side (mean -3 std) and excursions not as far away to the right side (mean + 1 std) were the important differences between the populations. A word length of about 4 to 7 was found to be the most successful. The largest magnitude t-score of -3.48 corresponds to *p*-value equal with 0.00125 for a two-tailed test and for degrees of freedom equal with 39. While the separation found between the two populations by this measure of entropy is considered statistically significant, the clinical significance of the measure identified here would have to be determined with additional experimentation.

The approximate entropy algorithm was also capable of detecting separation between the infants with typical development and the infants with delayed development. As with the symbolic entropy, the largest separations were seen between typical development and delayed development in the medial-lateral direction. Also, as with symbolic entropy, the larger t-scores for approximate entropy were negative, indicating that entropy calculated from postural sway data of infants with typical development is higher that entropy calculated from postural sway data of infants with delayed development. Overall, the best approximate entropy result (t-score = -3.48) was with lag = 4, m = 2, and r = 3*std. However several other combinations presented also larger values than the critical t value of 3.04, indicating significant differences between the two populations.

**Figure 3:**
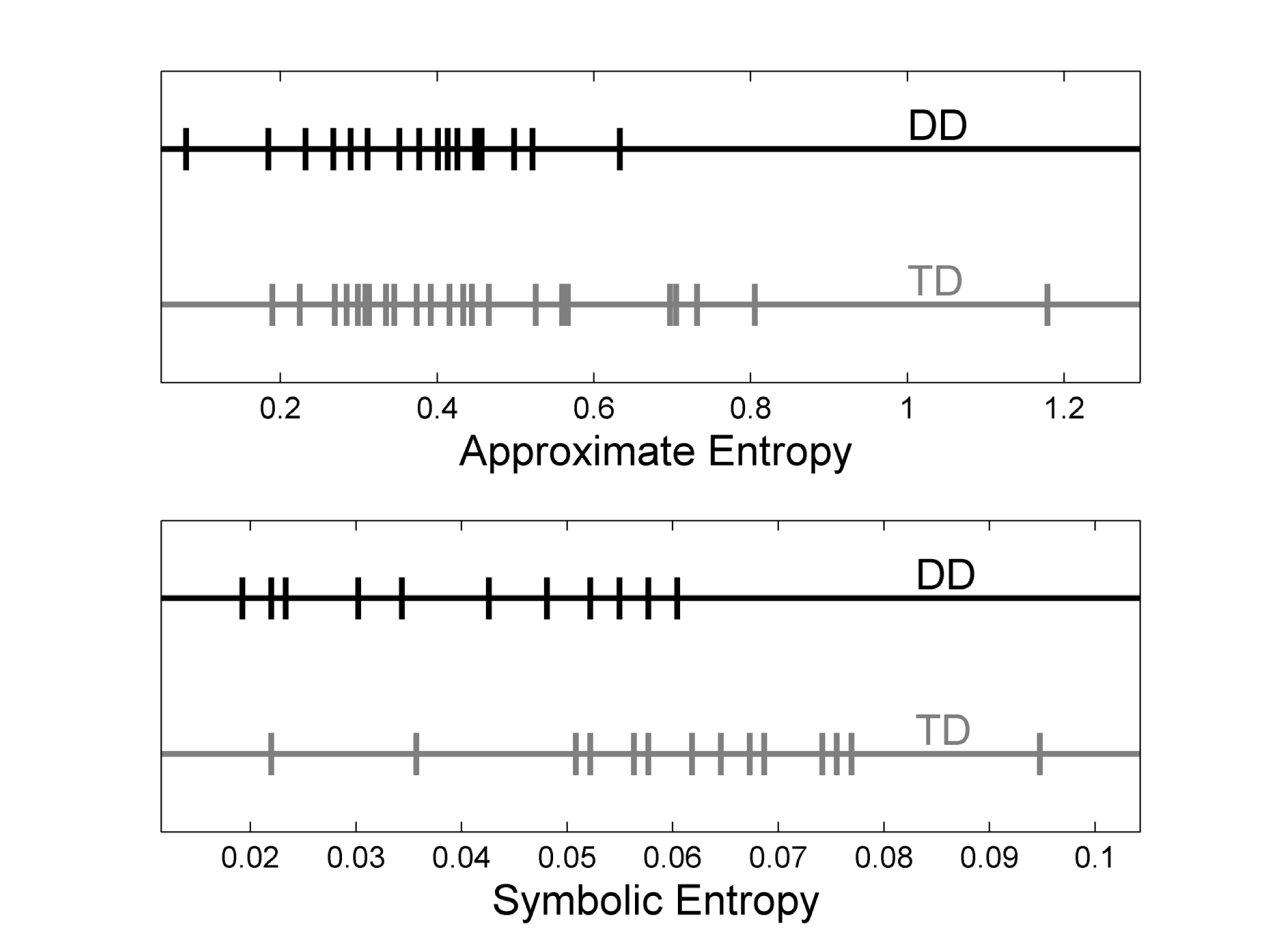
**Distribution of entropy values**. Distribution of entropy values for medial-lateral postural sway for infants who are typically developing versus those who have delayed development. Top plot (t-score = -1.94) is approximate entropy with r = 0.2 std, lag = 4, m = 2. Bottom plot (t-score = -3.48) is symbolic entropy with word length = 6, thresholds of -3 std and +1 std. Several of the subjects have the same symbolic entropy values as other subjects; the same number time series were analyzed for both top and bottom plots. The populations (DD and TD) are much better separated by use of symbolic entropy than approximate entropy.

In order to visually examine the effect of these parameters on the distribution of the entropy values, plots of the entropy values for the medial-lateral postural sway were calculated with two different methods (Fig 3). The top plot in Fig 3 shows the approximate entropy values that were obtained using the following parameters: m = 2, r = 0.2 std, and lag = 4. The bottom plot shows asymmetric symbolic entropy values that were obtained using two thresholds, mean – 3 std and mean + 1 std, and a word length of seven. This plot visually illustrates the benefit of using a method with a larger magnitude t-score for analysis of sitting postural sway in the medial-lateral direction to compare these two populations, as the populations can be seen to overlap quite a bit with the standard approximate entropy analysis (top) where as the separation is better in the asymmetric symbolic entropy analysis (bottom).

## Discussion

One aspect of this work was the exploration of the effects of various parameters in the entropy algorithms. While selection of the parameters used in the calculation of entropy was found to affect the results, the parameter values that give rise to statistically significant comparisons show consistent trends, with the typically developing infants having higher entropy values in sitting postural sway, and sway in the medial-lateral having the bigger differences between the populations.

Furthermore, two hypotheses were proposed in the introduction. One was that the complexity of postural sway of infants with delayed development would be altered as compared to that for infants with typical development. Importantly, a finding of this study was that the medial-lateral postural sway in sitting is a useful type of data to compare infants with delayed development with those who are typically developing, and that infants with typical development are seen to have more information entropy in their movement in this dimension than infants with delayed development, as measured by approximate entropy and symbolic entropy. This is consistent with the notion that development of a postural control strategy involves an exploration of the many possible solutions to Bernstein's degrees of freedom problem in order to arrive at a control strategy with optimal variability [7]. In this study we found that infants with typical development appear to be exploring more varied motor strategies, giving rise to a higher level of complexity in their postural sway. Therefore, healthy postural control is seen to be more complex as predicted by the optimal movement variability [7].

The second hypothesis, that lack of symmetry in anterior-posterior posterior control would be different between infants with delayed development and those with typical development, was not supported. A surprising result of this study was that the asymmetric symbolic entropy in the medial-lateral direction (left-right movement) found larger separation between postural sway in infants with developmental delay and those with typical development. We had expected this result in the anterior-posterior axis, since the result of a large excursion in the posterior direction is falling over, whereas a large excursion in the anterior direction merely results in the infant resting the torso on top of the legs. In fact, this was the motivation for trying the non-symmetric thresholds. However, the impact of the non-symmetric threshold was actually seen in the medial-lateral direction. As described in the experimental section, a researcher is always positioned to the left of the infant. Perhaps having a large object in the visual field unilaterally alters the infants' postural sway, as vision has been shown to impact standing postural sway in infants, although the effect was only seen in infants after walking skills had been acquired [31]. If integration of visual information is different in the two populations of infants, differences in postural sway could result. Alternatively, the non-symmetric postural sway may be due to some type of psychological response that the infants have to the presence of the adult on the left side, and this response is different in the two populations of infants. Infants develop a protective extension reaction [32], which is a reaction of the arms to falling from a seated position. The protective extension reaction develops first in the anterior direction, typically at around 6 months. Then it develops sideways, typically at around eight months. Finally, from about the tenth month, they are able to use their arms to prevent backwards falls. An infant who has developed this reaction for sideways falling may well respond differently to the presence of a researcher on one side than an infant who has not yet developed this reaction. Based on this typical development schedule of the protective extension reaction [32], we would expect that the typically developing infants would have developed this response, where as the infants with delayed development may not. However we did not test the infants for the protective extension response, so this is a speculative explanation. An alternative explanation which should be considered is that there may be some unconscious bias in how the researcher sitting next to the infants responds to near falls in the two populations, perhaps being more protective of falling movement away from themselves in infants that they perceive as having less control. The reason for the success of non-symmetric thresholding in the medial-lateral axis is not clear and warrants further investigation.

The results of this study indicate that optimization of the entropy algorithm for infant sitting postural sway data can greatly improve the ability to separate the infants with developmental delay from typically developing infants. However, there is still significant overlap of even the best entropy measures, which could result in false positives or false negatives if used in a clinical setting. Further improvements may be possible, such as optimization of the number of thresholds used in the calculation of symbolic entropy, optimization of the actual threshold values, and further exploration of non-symmetric thresholds. Additionally, there are other entropy algorithms that have not yet been applied to infant sitting postural sway data, which may offer an improvement. Multiscale entropy analysis [16] has been used on gait data [33] and on heart rate data [34]. Von Newman entropy, originally derived for quantum mechanics applications, has been applied to EEG data [35]. Kolmogorov entropy has been used on EEG data for epileptic seizure prediction [36] and on cell patch-clamp recordings [37]. Success in finding an algorithm that can objectively quantify pathologic motor patterns will help to identify infants who would benefit from therapeutic intervention, as well as provide an important research tool for assessment of various interventions for developmentally delayed infants.

Based on our exploration of different parameter combinations, we can make the following suggestions to researchers interested in using entropy measures in their work. Asymmetry can be an interesting aspect of postural sway data and of other time series data. However, asymmetry is not often probed, or if it is, then two separate force plates are required [38]. Use of the asymmetric symbolic entropy provides a means to investigate asymmetry on postural sway with data from a single force plate. Approximate entropy is a useful choice for an entropy measure, but the values for the parameters of m, lag, and r need to be optimized for the data set under investigation, rather than accepting standard values for these parameters.

## Conclusion

Information entropy measures can be used to characterize randomness in time series data. We have used approximate entropy and symbolic entropy in infant sitting postural sway for infants with typical development, and infants with delayed development, where the developmental delay was likely due to cerebral palsy. While selection of the parameters used in the calculation of entropy was found to affect the results, differences between the two populations found were to be consistent for statistically significant results. The significant results were that infants with typical development were found to have less repetition of fixed patterns in the medial-lateral direction of postural sway than infants with developmental delay. This result is consistent with the notion that infants with typical development are exploring a wider range of movement patterns as they learn to control upright sitting posture. This result also suggests that therapeutic interventions that encourage the exploration of varied movement patterns would be beneficial.

## Consent

Written consent for publication was obtained from the infant's parent (Figure 1).

## Competing interests

The authors declare that they have no competing interests.

## Authors' contributions

JED was involved with data collection, data analysis, and drafting of the manuscript. SLD was involved in data collection. RTH and AK were involved in data collection and subject recruiting. WAS and NS supervised the design and coordination of the study, and NS additionally supervised manuscript preparation. All authors read and approved the final manuscript.
